# Baseline immune-organ ^18^F-FDG PET/CT metabolism is associated with immune-related adverse events in anti-PD-1-treated non-small cell lung cancer

**DOI:** 10.3389/fmed.2026.1920274

**Published:** 2026-08-26

**Authors:** Haixu Zhu, Renhua Na, Xiaolong Yao, Jureti Azhati, Liming Chai, Canwen Sun, Bing Liu, Zeqiang Dai, Yan Xing

**Affiliations:** 1 Department of Nuclear Medicine, People's Hospital of Xinjiang Uyghur Autonomous Region, Urumqi, China; 2 Imaging Center, The First Affiliated Hospital of Xinjiang Medical University, Urumqi, China

**Keywords:** ^18^F-FDG PET/CT, anti-PD-1 therapy, immune-related adverse events, non-small cell lung cancer, spleen-to-liver ratio

## Abstract

**Objective:**

To investigate whether baseline ^18^F-FDG PET/CT immune-organ metabolic features are associated with immune-related adverse events (irAEs) in non-small cell lung cancer (NSCLC) patients receiving anti-PD-1 therapy.

**Methods:**

We retrospectively analyzed 120 stage III–IV NSCLC patients who underwent ^18^F-FDG PET/CT within 4 weeks before anti-PD-1 therapy and received ≥2 treatment cycles. The endpoint was any-grade irAE (CTCAE v5.0). Candidate predictors included tumor burden; spleen, bone-marrow, and thyroid metabolism; spleen-to-liver ratio (SLR); and bone-marrow-to-liver ratio. Standardized variables entered multivariable logistic regression with bootstrap optimism correction (1,000 resamples) and sensitivity analyses.

**Results:**

irAEs occurred in 66 patients (55.0%) after a median of 61.5 days; 18 (27.3%) had grade 3–4 events, mainly pneumonitis and hepatitis. Compared with non-irAE patients, irAE patients showed higher SLR, spleen SUVmax, bone-marrow indices, thyroid SUVmax, and neutrophil-to-lymphocyte ratio (NLR). In the final multivariable model, SLR (odds ratio [OR] 4.13 per 1-SD; 95% CI, 2.28–7.49; *p* < 0.001), thyroid SUVmax (OR 3.57; 95% CI, 1.96–6.49; *p* < 0.001), and NLR (OR 2.00; 95% CI, 1.24–3.24; *p* = 0.005) were independently associated with irAEs. Model AUC was 0.863 (optimism-corrected, 0.850), and persisted after excluding thyroiditis-first cases (AUC 0.874). Discrimination was lower when grade 3–4 events were used as the outcome (AUC 0.724), indicating that the model predicted any-grade irAEs more accurately than severe events.

**Conclusion:**

Baseline splenic and thyroid FDG metabolism, combined with systemic inflammation, were independently associated with irAEs in anti-PD-1-treated NSCLC, providing candidate pretreatment risk-stratification markers pending external validation.

## Introduction

Immune checkpoint inhibitors targeting programmed cell death protein 1 (PD-1) or its ligand have substantially transformed the treatment landscape of advanced non-small cell lung cancer (NSCLC), offering durable survival benefits across multiple therapeutic settings (1, 2). However, immune activation induced by these agents can result in immune-related adverse events (irAEs) affecting the skin, endocrine glands, lungs, gastrointestinal tract, liver, and other organs. Although many irAEs are mild or clinically manageable, severe toxicities may necessitate systemic corticosteroids, hospitalization, treatment interruption, or permanent discontinuation (3). Current clinical guidelines emphasize timely recognition and grade-adapted management, ranging from continued treatment with close monitoring for grade 1 events to treatment suspension, corticosteroid therapy, or permanent discontinuation for moderate-to-severe toxicities (4, 5).

Identifying patients at increased risk of irAEs before treatment initiation remains clinically important. Blood-based inflammatory biomarkers, such as the neutrophil-to-lymphocyte ratio (NLR), are readily available and may reflect systemic inflammatory status, but they provide only a limited representation of the host immune microenvironment (6, 7). By contrast, ^18^F-fluorodeoxyglucose positron emission tomography/computed tomography (^18^F-FDG PET/CT), which is routinely performed for staging and response assessment in NSCLC (8), may offer complementary information beyond tumor metabolic burden. Metabolic activity in immune-related or inflammation-prone organs—the spleen, bone marrow, and thyroid gland—can be quantified on baseline PET/CT and may serve as a non-invasive surrogate of systemic immune activation, hematopoietic response, or subclinical organ-specific inflammation. Standardized imaging protocols are critical, requiring weight-adjusted FDG administration, strict glucose control, and a ~ 60-min uptake period (9).

Additionally, emerging data suggest that immune-organ FDG uptake, notably the spleen-to-liver ratio (SLR), correlates with treatment efficacy and toxicity during immune checkpoint inhibition (10–12). Nevertheless, data from relatively homogeneous NSCLC cohorts remain limited, and the predictive value of baseline immune-organ metabolism for subsequent irAEs has not been fully clarified. Moreover, prediction-model studies are vulnerable to optimism when apparent performance is reported without transparent evaluation of calibration and internal validation. The TRIPOD statement and the updated TRIPOD+AI guidance highlight the need for explicit reporting of model development, discrimination, calibration, validation, and intended clinical use (13, 14).

Therefore, using individual-level data from a 120-patient NSCLC cohort, this study investigated whether baseline immune-organ metabolic parameters derived from ^18^F-FDG PET/CT, together with systemic inflammatory markers, were associated with the subsequent development of irAEs during anti-PD-1 therapy. We further aimed to develop a parsimonious exploratory prediction model to support hypothesis generation and provide a basis for future external validation.

## Materials and methods

### Ethics statement

This retrospective study was approved by the Institutional Review Board/Ethics Committee of People’s Hospital of Xinjiang Uyghur Autonomous Region (no. KY2026063004). The study was conducted in accordance with the Declaration of Helsinki. Owing to the retrospective nature of the study and the use of de-identified data, the requirement for written informed consent was waived by the ethics committee.

### Study design and patients

**Figure 1:**
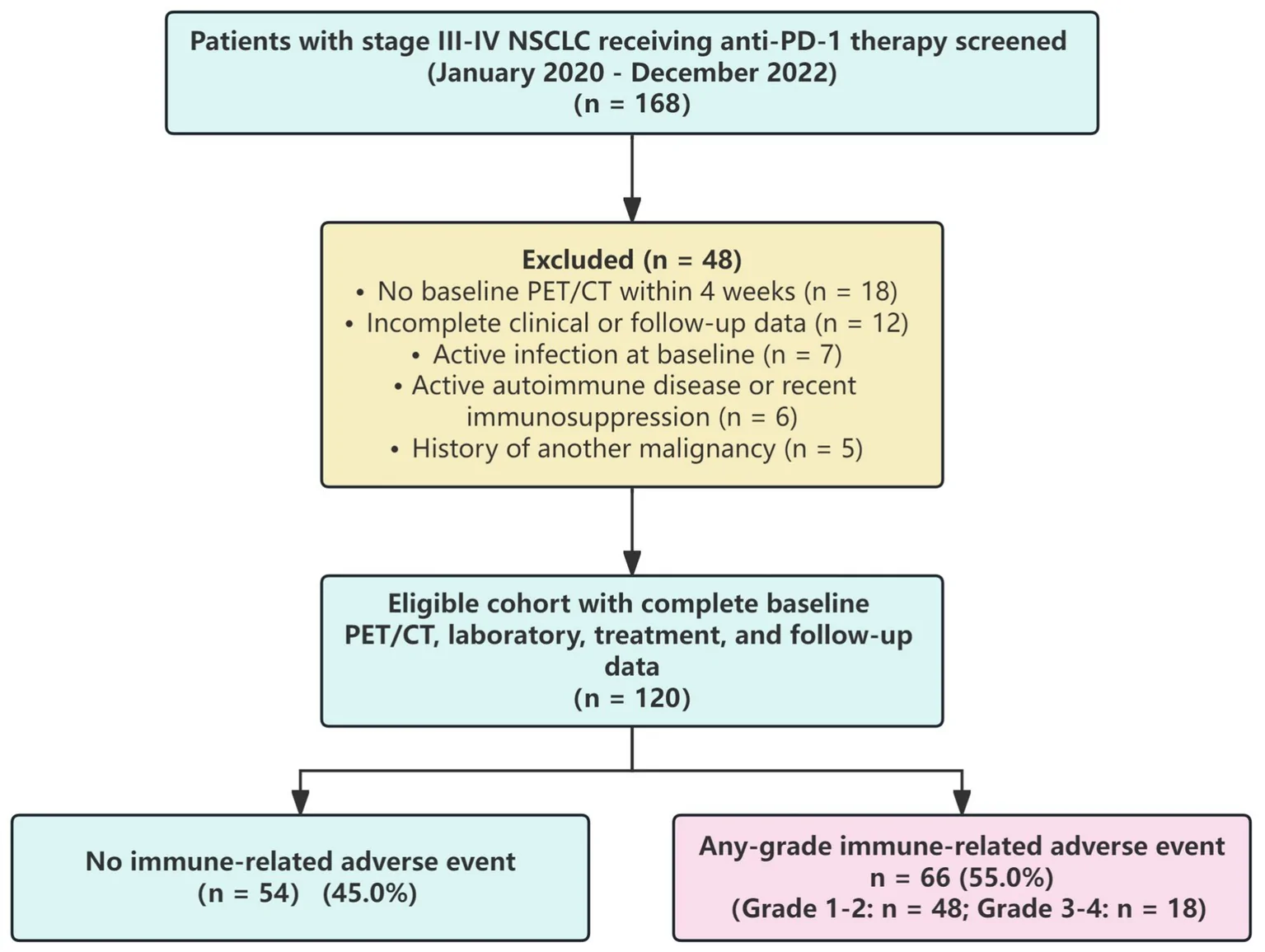
Patient flow diagram. Of 168 screened patients with stage III–IV NSCLC receiving anti-PD-1 therapy (January 2020–December 2022), 48 were excluded for prespecified reasons. The final analysis cohort comprised 120 patients (54 without irAEs and 66 with any-grade irAEs). NSCLC, non-small cell lung cancer; irAE, immune-related adverse event.

This retrospective single-center cohort study screened 168 consecutive patients with stage III–IV NSCLC who received anti-PD-1 therapy between January 2020 and December 2022. Patients were excluded if they had no baseline ^18^F-FDG PET/CT examination within 4 weeks before treatment initiation (*n* = 18), incomplete clinical or follow-up data (*n* = 12), active infection at baseline (*n* = 7), active autoimmune disease or systemic immunosuppressive therapy within 2 weeks before PET/CT (*n* = 6), or a history of another malignancy (*n* = 5). Ultimately, 120 patients were included in the final analysis (Figure 1). Eligible patients had histologically or cytologically confirmed NSCLC, underwent baseline ^18^F-FDG PET/CT before therapy, received at least two cycles of anti-PD-1 treatment, and had complete clinical, laboratory, imaging, treatment, and follow-up data. The study was reported in accordance with the TRIPOD and TRIPOD+AI recommendations (13, 14).

### Treatment and clinical variables

Anti-PD-1 therapy consisted of sintilimab, pembrolizumab, tislelizumab, or nivolumab, administered as monotherapy or combined with chemotherapy. Collected clinical variables included age, sex, body mass index, smoking history, Eastern Cooperative Oncology Group (ECOG) performance status, histologic subtype, TNM stage, PD-L1 tumor proportion score, driver mutation status, anti-PD-1 agent, treatment regimen, treatment line, and baseline laboratory indices. Laboratory data were obtained within 7 days before anti-PD-1 initiation and within 14 days of baseline PET/CT, before corticosteroids, antibiotics, or other acute interventions whenever possible. The NLR was calculated by dividing the absolute neutrophil count by the absolute lymphocyte count.

### Endpoint definition

The primary endpoint was the occurrence of any-grade irAE during anti-PD-1 therapy, graded according to the Common Terminology Criteria for Adverse Events version 5.0. Any-grade irAE was prespecified as the primary endpoint for two reasons: first, the recognition of any immune toxicity—not only high-grade events—triggers guideline-directed changes in monitoring and management, so that early low-grade events are themselves clinically actionable; and second, the higher frequency of the composite endpoint provided the event count required for stable multivariable modeling in this single-center cohort. Grade 3–4 irAEs, which are less frequent but more clinically consequential, were analysed separately as a secondary outcome (see Statistical analysis). irAEs were identified through review of clinical notes, laboratory results, imaging reports, treatment interruptions, corticosteroid use, and specialist consultations. The assessment window extended from anti-PD-1 initiation to the last follow-up or death; events occurring within 90 days after the final dose were also included if considered immune-mediated. For each patient, irAE type, highest grade, and time to first irAE were recorded. irAE attribution and grading were independently reviewed by two oncologists blinded to PET/CT quantitative results, with disagreements resolved by consensus with a senior oncologist.

The secondary endpoint was overall survival (OS), defined as the interval from anti-PD-1 initiation to death from any cause or last follow-up. Because OS comparisons by ever-irAE status are susceptible to immortal-time bias, survival analyses were considered exploratory and supplemented by a 3-month landmark analysis.

### PET/CT acquisition and image analysis

Patients fasted for at least 6 h before intravenous administration of ^18^F-FDG (3.7–5.5 MBq/kg), and blood glucose was confirmed to be <11.1 mmol/L before injection. PET/CT was performed approximately 60 min after tracer administration (acceptable range, 55–75 min), using an integrated PET/CT scanner (GE Discovery PET/CT 710, GE Healthcare) after routine quality control. Imaging covered the skull base to mid-thigh. Low-dose CT was obtained for attenuation correction and anatomic localization (120 kVp, automatic tube-current modulation, 3.75–5.0-mm reconstructed slice thickness). PET emission data were acquired for 2–3 min per bed position and reconstructed using ordered-subset expectation maximization with CT-based attenuation, scatter, and random corrections. These conditions were consistent with international oncologic PET/CT procedure recommendations (9).

Images were independently reviewed by two experienced nuclear medicine physicians (each with more than 5 years of PET/CT reporting experience) blinded to irAE outcomes, laboratory indices, and survival status. Primary tumor SUVmax and SUVmean were measured on attenuation-corrected PET. Whole-body metabolic tumor volume (MTV) was calculated using a 40% SUVmax threshold, and total lesion glycolysis (TLG) as the sum of lesion-level MTV × SUVmean, in line with PERCIST principles (15). Liver SUVmean was measured using a 3-cm spherical region of interest (ROI) in homogeneous right hepatic parenchyma. Spleen SUVmax was obtained from a 2–3-cm spherical ROI placed in central splenic parenchyma. Bone-marrow SUVmax was the highest SUVmax among spherical ROIs placed centrally in lumbar vertebrae L1–L5. Thyroid SUVmax was measured in both lobes while avoiding focal nodules and spillover, with the higher value recorded. SLR was calculated as spleen SUVmax / liver SUVmean, and BLR as bone-marrow SUVmax / liver SUVmean. In a random 30-patient subset, interobserver agreement was good to excellent (intraclass correlation coefficients >0.85). Interobserver agreement was assessed separately for each key metric—spleen SUVmax, SLR, thyroid SUVmax, liver SUVmean, and bone-marrow SUVmax—and the intraclass correlation coefficient exceeded 0.85 for every parameter, supporting the between-reader reproducibility of the quantitative indices used in the model.

### Statistical analysis

Continuous variables were summarized as medians with interquartile ranges and compared using the Mann–Whitney U test. Categorical variables were summarized as counts and percentages and compared using the chi-square test or Fisher’s exact test. Logistic regression was used to evaluate predictors of any-grade irAEs, with continuous predictors standardized and reported per 1-standard-deviation (SD) increase. Variables with univariable *p* < 0.10, together with clinically relevant PET/CT indices, were considered for multivariable modeling. The SLR was prioritized over spleen SUVmax because the liver-normalized ratio was prespecified as the principal spleen-related marker. The minimum sample-size considerations for multivariable prediction models with binary outcomes were taken into account when selecting the maximum number of candidate predictors (16). Multicollinearity was assessed using variance inflation factors.

Discrimination was evaluated using the area under the receiver operating characteristic curve (AUC); cutoffs were selected using the Youden index. AUC 95% confidence intervals were estimated by nonparametric bootstrap resampling. Internal validation was performed using 1,000 bootstrap resamples, with the same candidate-predictor pool and final model-fitting procedure repeated in each resample. Optimism was estimated as the mean difference between bootstrap-model performance in the bootstrap sample and performance in the original sample, and was applied to AUC correction. Calibration was assessed using calibration plots, apparent Brier score, apparent calibration slope, and the Hosmer–Lemeshow test. The full multivariable model equation, regression coefficients, and standardization parameters are presented in the results section to support reproducibility, in accordance with TRIPOD recommendations (13, 14).

Sensitivity analyses included excluding patients whose first documented irAE was thyroiditis, using grade 3–4 irAE as the outcome, adjusting for age, sex, and ECOG performance status, and additional *post hoc* adjustment for treatment regimen and treatment line. OS was estimated using the Kaplan–Meier method and compared using the log-rank test; a 3-month landmark analysis with Cox regression was performed to reduce immortal-time bias. All tests were two-sided, and *p* < 0.05 was considered statistically significant.

## Results

### Patient characteristics

Among 168 screened patients, 120 were included in the final analysis (Figure 1). The cohort included 95 men (79.2%) and 25 women (20.8%), with a median age of 64 years (interquartile range [IQR], 58–71). irAEs occurred in 66 patients (55.0%), with a median time to onset of 61.5 days (IQR, 47.0–92.5; range, 16–155 days). Baseline clinical characteristics were generally comparable between the irAE and non-irAE groups (Table 1). The NLR was higher in patients who developed irAEs, whereas other baseline laboratory parameters were not significantly different between groups (Table 2).

**Table 1:** Baseline clinical characteristics according to irAE status.

Characteristic	Total (*N* = 120)	Non-irAEs (*n* = 54)	irAEs (*n* = 66)	*p* value
Age, years	64 (58–71)	66 (61–72)	62 (57–71)	0.159
Body mass index, kg/m^2^	23.4 (22.2–25.8)	23.6 (22.6–25.8)	23.4 (21.9–25.8)	0.786
PD-L1 TPS, %	34 (2–67)	37 (8–65)	30 (1–67)	0.924
Sex, male/female	95 (79.2)/25 (20.8)	39 (72.2)/15 (27.8)	56 (84.8)/10 (15.2)	0.090
Smoking history, yes/no	52 (43.3)/68 (56.7)	27 (50.0)/27 (50.0)	25 (37.9)/41 (62.1)	0.183
ECOG PS, 0/1/2	17 (14.2)/79 (65.8)/24 (20.0)	8 (14.8)/34 (63.0)/12 (22.2)	9 (13.6)/45 (68.2)/12 (18.2)	0.821
Histology, Ad/SCC	57 (47.5)/63 (52.5)	24 (44.4)/30 (55.6)	33 (50.0)/33 (50.0)	0.544
TNM stage, III/IV	36 (30.0)/84 (70.0)	13 (24.1)/41 (75.9)	23 (34.8)/43 (65.2)	0.200
PD-L1 TPS, <1%/1–49%/≥50%	15 (12.5)/60 (50.0)/45 (37.5)	9 (16.7)/27 (50.0)/18 (33.3)	6 (9.1)/33 (50.0)/27 (40.9)	0.403
Driver mutation, yes/no	16 (13.3)/104 (86.7)	10 (18.5)/44 (81.5)	6 (9.1)/60 (90.9)	0.131
Anti-PD-1 agent				0.856
Sintilimab	31 (25.8)	15 (27.8)	16 (24.2)	
Pembrolizumab	31 (25.8)	13 (24.1)	18 (27.3)	
Tislelizumab	26 (21.7)	13 (24.1)	13 (19.7)	
Nivolumab	32 (26.7)	13 (24.1)	19 (28.8)	
Treatment regimen, CT/mono	89 (74.2)/31 (25.8)	41 (75.9)/13 (24.1)	48 (72.7)/18 (27.3)	0.690
Treatment line, 1/2/3	67 (55.8)/34 (28.3)/19 (15.8)	28 (51.9)/20 (37.0)/6 (11.1)	39 (59.1)/14 (21.2)/13 (19.7)	0.117

Data are medians (IQRs) or *n* (%). *p* values were derived from Mann–Whitney U tests (continuous variables) or chi-square/Fisher exact tests (categorical variables) and were not adjusted for multiple comparisons. Ad, adenocarcinoma; CT, combination chemotherapy; ECOG PS, Eastern Cooperative Oncology Group performance status; irAE, immune-related adverse event; mono, monotherapy; PD-1, programmed cell death protein 1; PD-L1, programmed death-ligand 1; SCC, squamous cell carcinoma; TPS, tumor proportion score.

**Table 2:** Baseline laboratory and PET/CT metabolic parameters according to irAE status.

Characteristic	Total (*N* = 120)	Non-irAEs (*n* = 54)	irAEs (*n* = 66)	*p* value
NLR	5.17 (3.46–8.17)	4.56 (3.07–7.15)	5.62 (3.93–9.02)	0.033
LDH, U/L	224.2 (203.2–247.1)	223.4 (199.4–253.4)	225.8 (205.0–240.3)	0.506
Albumin, g/L	38.4 (35.3–40.9)	38.7 (35.8–40.3)	38.2 (35.3–41.4)	0.996
CRP, mg/L	11.49 (6.32–25.04)	11.66 (7.51–20.28)	11.38 (5.79–26.99)	0.856
WBC, ×10^9^/L	7.50 (5.95–8.57)	7.60 (5.53–8.59)	7.48 (6.21–8.50)	0.829
Neutrophil count, ×10^9^/L	5.88 (4.12–6.89)	5.80 (3.62–6.75)	5.96 (4.57–6.94)	0.506
Lymphocyte count, ×10^9^/L	1.08 (0.74–1.30)	1.12 (0.85–1.36)	1.04 (0.68–1.25)	0.065
Primary SUVmax	13.5 (9.4–20.1)	13.7 (10.4–21.5)	12.9 (9.2–18.4)	0.373
Primary SUVmean	8.2 (5.6–11.5)	8.5 (6.3–11.6)	7.5 (5.3–11.2)	0.338
Whole-body MTV, cm^3^	63.3 (43.0–107.2)	74.4 (52.7–109.9)	58.7 (38.1–86.9)	0.034
Whole-body TLG	254.3 (142.5–436.8)	294.5 (163.0–473.5)	234.2 (136.2–392.0)	0.085
Liver SUVmean	2.69 (2.34–3.03)	2.72 (2.44–3.11)	2.58 (2.26–2.98)	0.184
Spleen SUVmax	2.49 (2.16–3.02)	2.24 (2.06–2.52)	2.84 (2.44–3.22)	<0.001
SLR	0.98 (0.79–1.19)	0.82 (0.72–0.99)	1.12 (0.94–1.29)	<0.001
Bone-marrow SUVmax	2.58 (2.23–2.99)	2.44 (2.19–2.81)	2.67 (2.28–3.14)	0.014
BLR	0.98 (0.78–1.21)	0.92 (0.72–1.10)	1.08 (0.86–1.25)	0.006
Thyroid SUVmax	2.04 (1.71–2.47)	1.83 (1.45–2.17)	2.15 (1.83–2.70)	<0.001

Data are medians (IQRs). *p* values were derived from Mann–Whitney U tests and were not adjusted for multiple comparisons. BLR, bone-marrow-to-liver ratio; CRP, C-reactive protein; LDH, lactate dehydrogenase; MTV, metabolic tumor volume; NLR, neutrophil-to-lymphocyte ratio; SLR, spleen-to-liver ratio; SUV, standardized uptake value; TLG, total lesion glycolysis; WBC, white blood cell count.

### Baseline PET/CT metabolic parameters and irAEs

**Figure 2:**
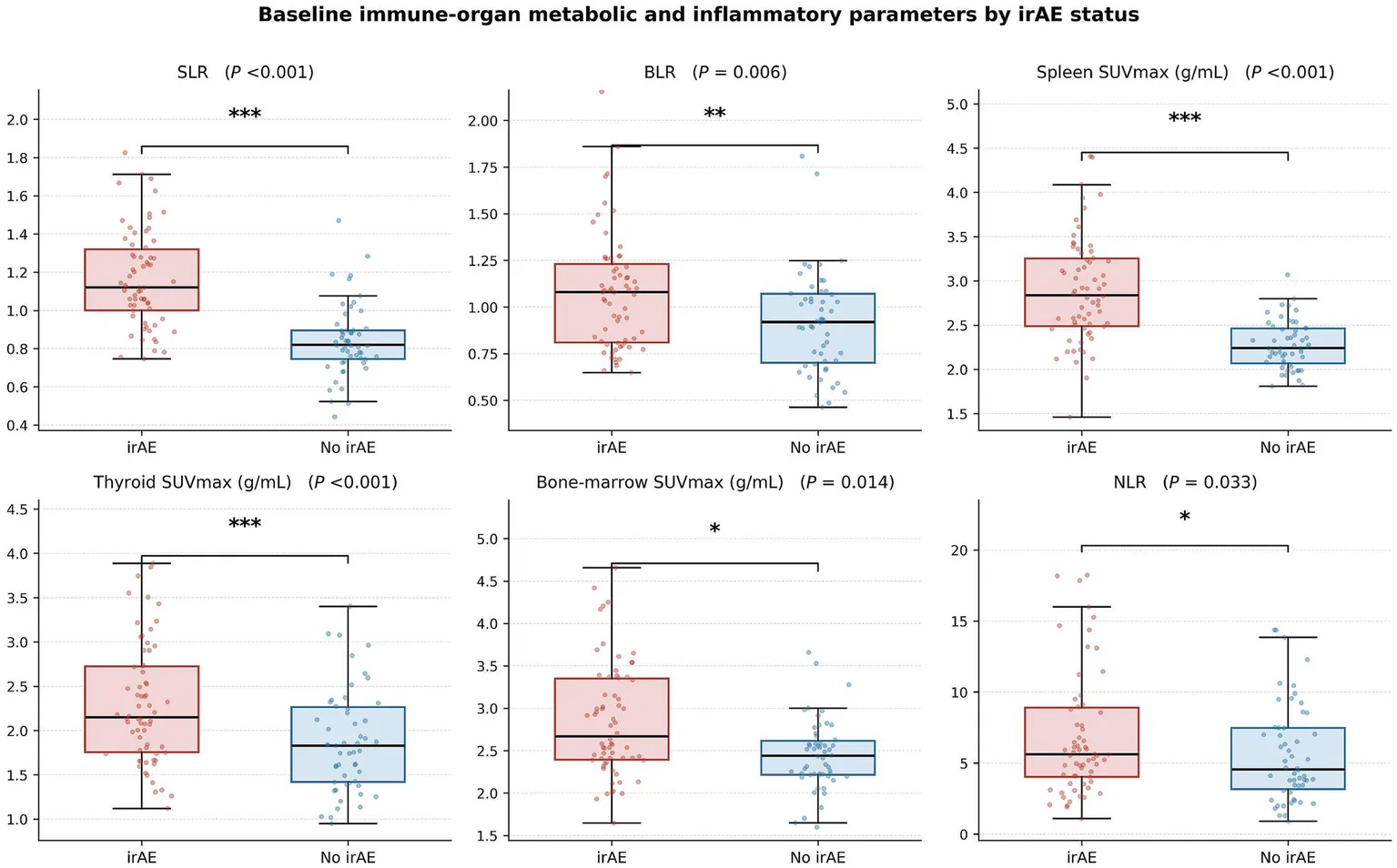
Baseline immune-organ metabolic and inflammatory parameters by irAE status. Boxplots with overlaid individual data points compare patients with irAEs (red, *n* = 66) and without irAEs (blue, *n* = 54). Boxes represent the interquartile range, horizontal lines within boxes denote medians, whiskers extend to 1.5 × IQR, and open circles indicate outliers. *p* values are from Mann–Whitney U tests. ***p < 0.001; **p < 0.01; *p < 0.05. BLR, bone-marrow-to-liver ratio; NLR, neutrophil-to-lymphocyte ratio; SLR, spleen-to-liver ratio; SUV, standardized uptake value (g/mL).

Patients who developed irAEs showed higher baseline spleen-related, bone-marrow–related, and thyroid metabolic activity than those without irAEs (Figure 2; Table 2). The SLR was significantly higher in the irAE group (median, 1.12 [IQR, 0.94–1.29] vs. 0.82 [0.72–0.99]; *p* < 0.001), with similar increases observed for spleen SUVmax, BLR, bone-marrow SUVmax, and thyroid SUVmax (all *p* < 0.05). Conversely, whole-body MTV was lower among patients who developed irAEs (*p* = 0.034). Primary tumor uptake and liver SUVmean did not differ significantly between groups.

### Multivariable associations with irAEs

In univariable analyses, spleen SUVmax, SLR, and thyroid SUVmax showed the strongest associations with irAEs (Table 3). In the final multivariable model, SLR, thyroid SUVmax, and NLR remained independently associated with any-grade irAEs: SLR (OR per 1-SD increase, 4.13; 95% CI, 2.28–7.49; *p* < 0.001), thyroid SUVmax (OR, 3.57; 95% CI, 1.96–6.49; *p* < 0.001), and NLR (OR, 2.00; 95% CI, 1.24–3.24; *p* = 0.005) (Table 4). No meaningful multicollinearity was observed (all variance inflation factors ≤1.07).

**Table 3:** Univariable logistic regression for any-grade irAEs.

Variable	Scale	OR (95% CI)	*p* value
Spleen SUVmax	per 1-SD increase	3.19 (1.93–5.26)	<0.001
SLR	per 1-SD increase	3.01 (1.84–4.94)	<0.001
Thyroid SUVmax	per 1-SD increase	2.05 (1.34–3.13)	<0.001
Bone-marrow SUVmax	per 1-SD increase	1.69 (1.13–2.52)	0.010
BLR	per 1-SD increase	1.66 (1.11–2.47)	0.014
Lymphocyte count	per 1-SD increase	0.69 (0.47–1.00)	0.053
NLR	per 1-SD increase	1.41 (0.96–2.09)	0.083
Whole-body TLG	per 1-SD increase	0.72 (0.50–1.06)	0.093
Whole-body MTV	per 1-SD increase	0.74 (0.51–1.08)	0.121
CRP	per 1-SD increase	1.38 (0.91–2.10)	0.131
Liver SUVmean	per 1-SD increase	0.78 (0.54–1.12)	0.181
Primary SUVmean	per 1-SD increase	0.83 (0.58–1.19)	0.316
Primary SUVmax	per 1-SD increase	0.84 (0.58–1.20)	0.342
Neutrophil count	per 1-SD increase	1.19 (0.83–1.70)	0.356
LDH	per 1-SD increase	0.85 (0.59–1.22)	0.388
WBC	per 1-SD increase	1.10 (0.76–1.57)	0.619
Albumin	per 1-SD increase	1.02 (0.71–1.46)	0.928

All continuous predictors were standardized (mean = 0, SD = 1) before regression. Variables are ranked by ascending *p* value. OR > 1 indicates higher odds of irAE per 1-SD increase in the predictor. BLR, bone-marrow-to-liver ratio; CI, confidence interval; CRP, C-reactive protein; irAE, immune-related adverse event; LDH, lactate dehydrogenase; MTV, metabolic tumor volume; NLR, neutrophil-to-lymphocyte ratio; OR, odds ratio; SD, standard deviation; SLR, spleen-to-liver ratio; SUV, standardized uptake value; TLG, total lesion glycolysis; WBC, white blood cell count.

**Table 4:** Final multivariable logistic regression model for any-grade irAEs.

Variable	OR (95% CI)	*p* value	β coefficient
SLR, per 1-SD increase	4.13 (2.28–7.49)	<0.001	1.42
Thyroid SUVmax, per 1-SD increase	3.57 (1.96–6.49)	<0.001	1.27
NLR, per 1-SD increase	2.00 (1.24–3.24)	0.005	0.69

Regression coefficients (β) were calculated from the final logistic model using standardized predictors. Standardization parameters: SLR mean = 1.012, SD = 0.276; thyroid SUVmax mean = 2.057, SD = 0.613; NLR mean = 6.365, SD = 3.917. Model intercept = 0.383. Hosmer-Lemeshow goodness-of-fit was assessed to confirm adequate model calibration. CI, confidence interval; NLR, neutrophil-to-lymphocyte ratio; OR, odds ratio; SD, standard deviation; SLR, spleen-to-liver ratio; SUV, standardized uptake value.

For reproducibility, the full final model can be expressed as: logit(p) = 0.38 + 1.42 × z_SLR + 1.27 × z_Thyroid SUVmax + 0.69 × z_NLR, where z_SLR = (SLR − 1.012)/0.276, z_Thyroid SUVmax = (thyroid SUVmax − 2.057)/0.613, and z_NLR = (NLR − 6.365)/3.917. The predicted probability is *p* = 1 / [1 + exp.(−logit(p))].

### Model performance, calibration, and sensitivity analyses

**Figure 3:**
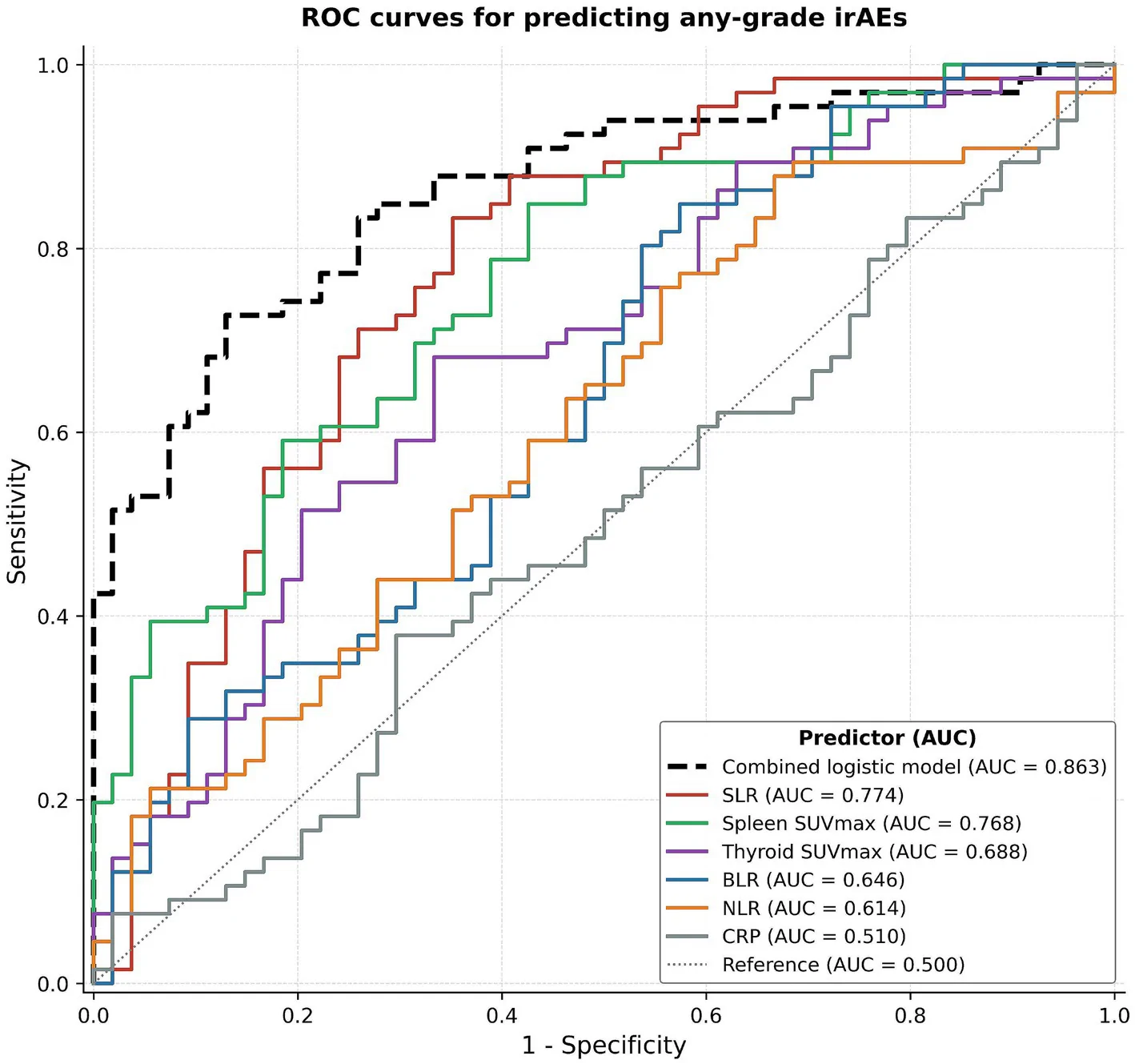
ROC curves for predicting any-grade irAEs. ROC curves and AUCs for individual baseline markers and the combined three-variable logistic model (SLR + thyroid SUVmax + NLR; black dashed line). The dotted diagonal represents a non-informative reference (AUC = 0.500). AUC, area under the curve; CRP, C-reactive protein; ROC, receiver operating characteristic. Other abbreviations as in Figure 2.

**Figure 4:**
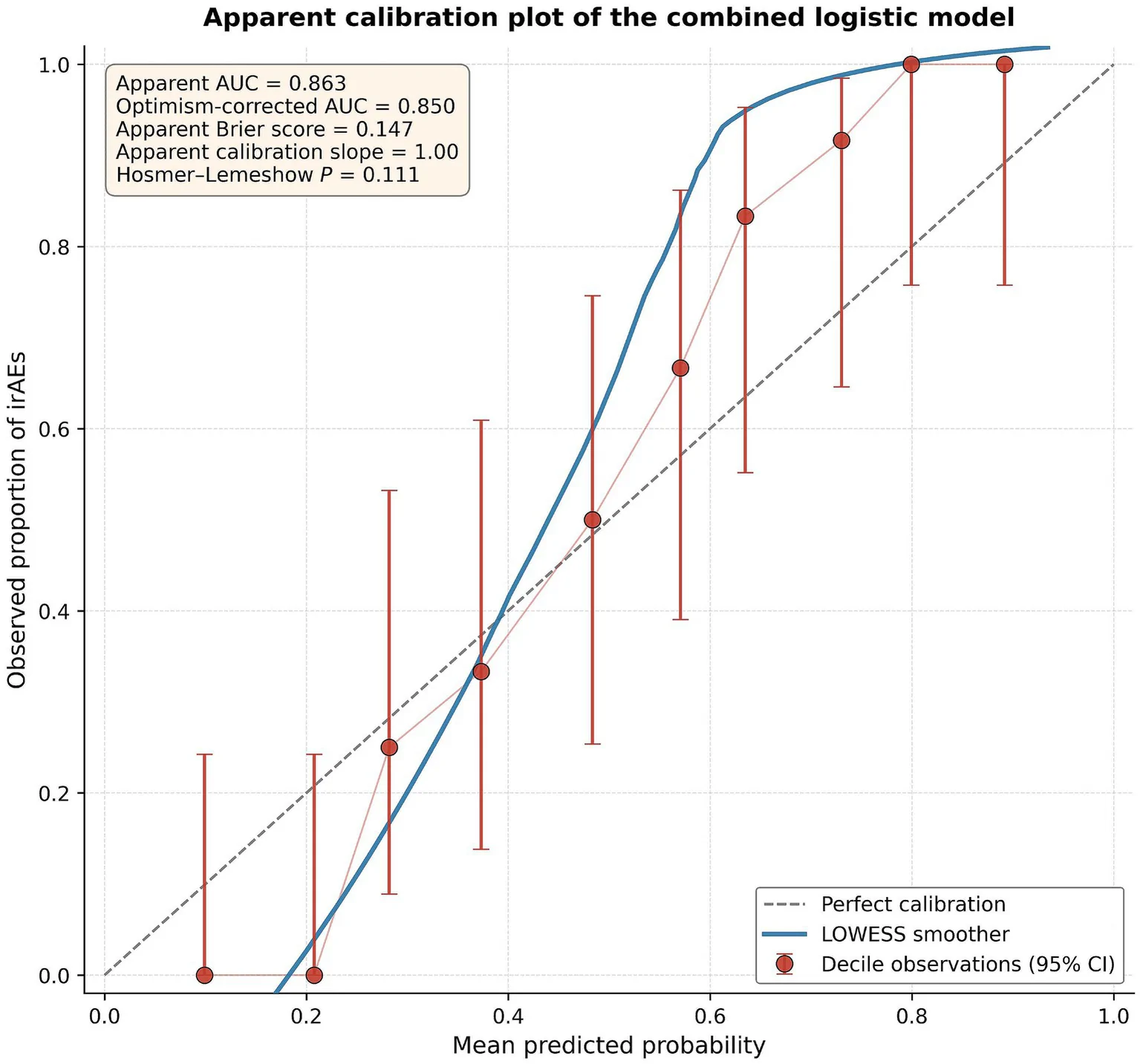
Apparent calibration plot of the combined logistic model. Predicted probabilities were grouped into deciles. Red points denote observed event proportions per decile with Wilson 95% confidence intervals; the blue line is a LOWESS smoother fitted to individual-level data; the gray dashed line indicates perfect calibration. Apparent model performance metrics are shown in the inset.

Among the individual markers, SLR showed the highest discrimination (AUC, 0.774; bootstrap 95% CI, 0.688–0.859), followed by spleen SUVmax, thyroid SUVmax, BLR, and NLR (Figure 3; Table 5). The combined logistic model further improved discrimination, yielding an apparent AUC of 0.863 and an optimism-corrected AUC of 0.850. Calibration in the development cohort showed a Brier score of 0.147, a calibration slope of 1.00, and a non-significant Hosmer–Lemeshow test (*p* = 0.111) (Figure 4). Sensitivity analyses confirmed the robustness of the findings: model performance remained similar after exclusion of thyroiditis-first irAEs (AUC, 0.874), after adjustment for age, sex, and ECOG performance status (AUC, 0.875), and after further *post hoc* adjustment for treatment regimen and treatment line (AUC, 0.862), whereas discrimination was attenuated when grade 3–4 irAEs were used as the outcome (AUC, 0.724) (Supplementary Table S1).

**Table 5:** Discrimination of individual markers and the combined model.

Marker/model	AUC (95% CI)	Cutoff	Sens, %	Spec, %	TP	FP	TN	FN
SLR	0.774 (0.688–0.859)	0.91	81.8	68.5	54	17	37	12
Spleen SUVmax	0.768 (0.681–0.849)	2.67	66.7	81.5	44	10	44	22
Thyroid SUVmax	0.688 (0.590–0.778)	2.01	68.2	64.8	45	19	35	21
BLR	0.646 (0.544–0.744)	0.97	65.2	59.3	43	22	32	23
NLR	0.614 (0.512–0.711)	3.89	75.8	46.3	50	29	25	16
CRP	0.510 (0.406–0.614)	34.63	22.7	94.4	15	3	51	51
Combined model	0.863 (0.788–0.928)	0.41	89.4	70.4	59	16	38	7

Cutoffs were determined by the Youden index. Sensitivity, specificity, and classification counts were derived from the same development cohort without optimism correction or external validation; they describe apparent performance only and should not be used for clinical decision-making. AUC, area under the receiver operating characteristic curve; BLR, bone-marrow-to-liver ratio; CI, confidence interval; CRP, C-reactive protein; FN, false negative; FP, false positive; NLR, neutrophil-to-lymphocyte ratio; Sens, sensitivity; SLR, spleen-to-liver ratio; Spec, specificity; SUV, standardized uptake value; TN, true negative; TP, true positive.

### irAE profile and exploratory survival analysis

**Figure 5:**
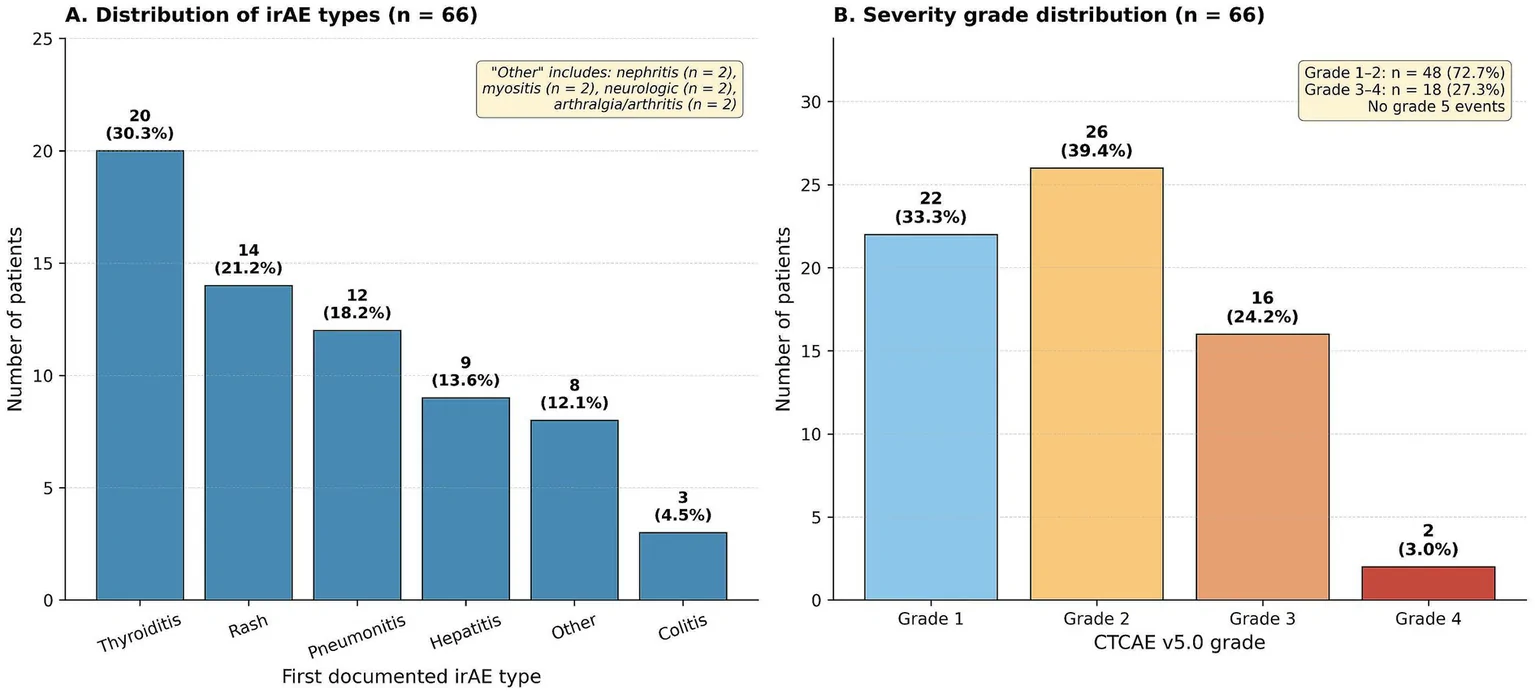
Distribution of irAE types and severity grades among the 66 patients with irAEs **(A)** First documented irAE types. The “Other” category included nephritis, myositis, neurologic events, and arthralgia/arthritis (*n* = 2 each). **(B)** Severity grades per CTCAE v5.0; no grade 5 events occurred. CTCAE, Common Terminology Criteria for Adverse Events.

The distribution of irAE types and severity grades among the 66 patients with irAEs is shown in Figure 5. Among these, 48 (72.7%) experienced grade 1–2 events and 18 (27.3%) experienced grade 3–4 events. The most frequent severe (grade ≥3) irAEs were immune-related pneumonitis, hepatitis, and colitis. Thyroiditis was the most common first irAE (*n* = 20, 30.3%), followed by rash, pneumonitis, and hepatitis. No grade 5 irAEs were observed; one patient discontinued therapy permanently due to grade 4 pneumonitis.

**Figure 6:**
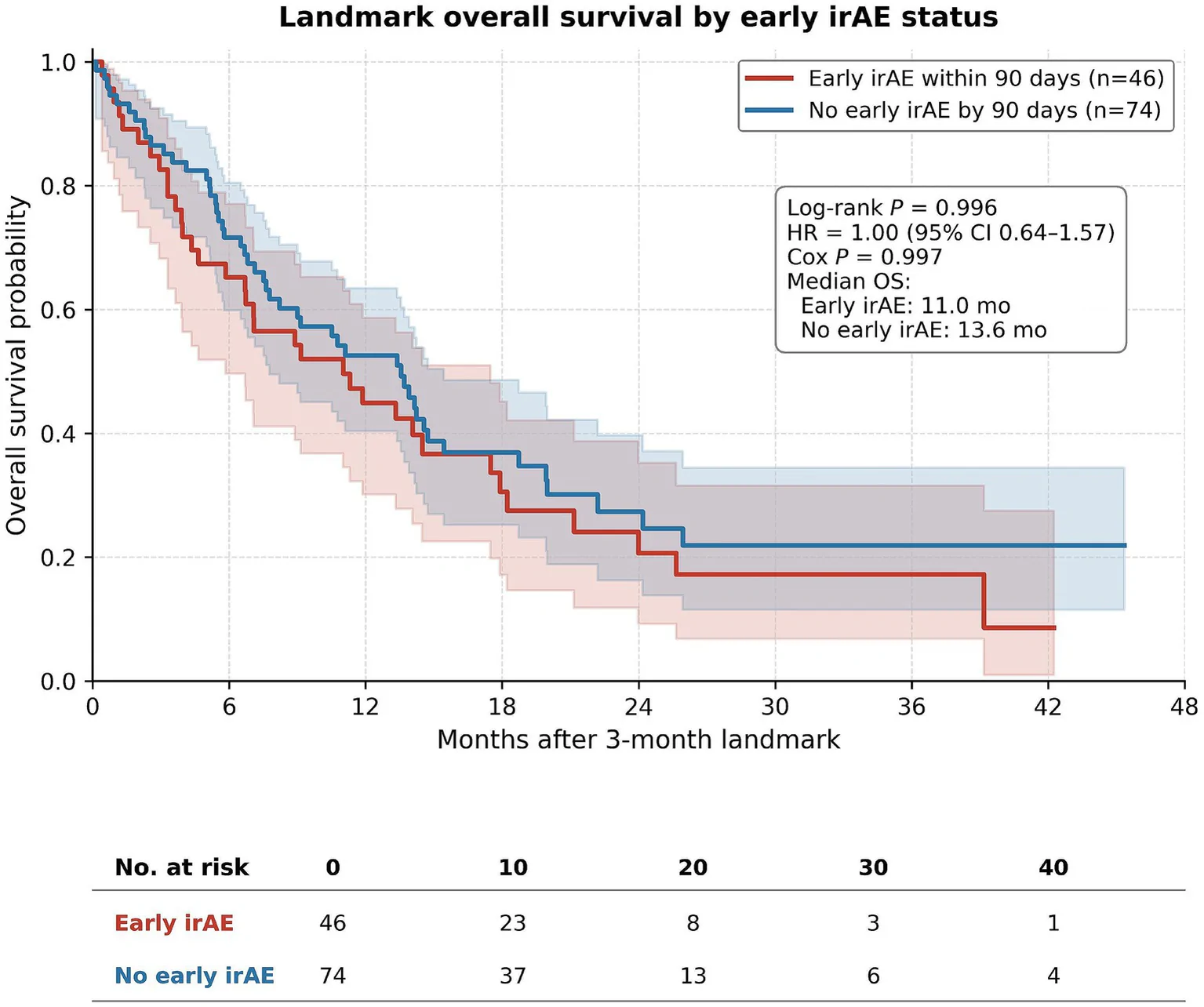
Landmark overall survival by early irAE status. Kaplan–Meier estimates of overall survival using a 3-month landmark analysis, with the time origin reset to 90 days after anti-PD-1 initiation. Patients were classified as having early irAE within 90 days (red, *n* = 46) or not (blue, *n* = 74). Shaded bands represent 95% confidence intervals. The number-at-risk table is shown below the curves. HR, hazard ratio.

During a median follow-up of 15.0 months (IQR, 10.4–18.0), 81 deaths occurred and 39 patients were censored. No patient died or was lost to follow-up before the 3-month landmark, and all 120 patients were therefore included in the landmark analysis. OS was similar between patients with and without early irAEs (log-rank *p* = 0.996; hazard ratio, 1.00; 95% CI, 0.64–1.57; *p* = 0.997), indicating no detectable association between early irAEs and OS in this cohort after landmark correction (Figure 6).

## Discussion

In this individual-level analysis of patients with stage III–IV NSCLC receiving anti-PD-1 therapy, baseline immune-organ metabolic activity—particularly the SLR and thyroid SUVmax—was independently associated with subsequent irAEs. Incorporating the NLR, a routinely available marker of systemic inflammation, further enhanced multivariable prediction. The final three-variable model demonstrated favorable apparent discrimination with limited bootstrap optimism, supporting the concept that pre-treatment host immune tone—captured jointly through PET/CT and peripheral blood—can stratify patients by toxicity risk (13, 14).

The biological plausibility of SLR as a toxicity marker is well supported by established splenic immunology. As a secondary lymphoid organ central to antigen presentation, lymphocyte trafficking, and systemic inflammatory regulation, the spleen reflects baseline immune activation status; consequently, higher splenic FDG uptake normalized to liver background may indicate a host immune tone predisposed to both therapeutic immune activation and off-target autoimmunity. Recent PET/CT studies have linked splenic and other secondary lymphoid organ metabolic activity to outcomes during checkpoint blockade (17, 18). Our findings extend this concept to a homogeneous anti-PD-1-treated NSCLC population, demonstrating that SLR remains independently associated with irAEs after adjustment for thyroid uptake and NLR.

Several biological mechanisms may underlie the link between elevated baseline splenic FDG uptake and subsequent irAEs. Increased splenic glucose metabolism likely reflects systemic activation of both adaptive and innate immune compartments—including expansion of clonal T-cell pools, heightened myeloid trafficking, and enhanced antigen-presenting cell turnover—all of which prime the host for a more robust, and potentially less self-tolerant, response upon PD-1 blockade (19). Splenic myeloid-derived suppressor cells also modulate peripheral self-reactivity, providing a mechanistic link between baseline splenic immune tone and downstream off-target inflammation (20). Increased thyroid FDG uptake may similarly represent subclinical lymphocytic infiltration that is unmasked clinically once PD-1–mediated peripheral tolerance is removed; recent prospective data have shown that elevated thyroidal FDG activity during ICI therapy temporally precedes biochemical and clinical hypothyroidism (21). Together, these observations support the concept that baseline ^18^F-FDG PET/CT captures a “primed immune state”—at both organ-specific (thyroid) and systemic (spleen) levels—that confers higher susceptibility to irAEs once checkpoint inhibition is initiated.

Thyroid SUVmax represents a second mechanistically meaningful predictor. Endocrine irAEs—particularly thyroid dysfunction and thyroiditis—are among the most common toxicities during PD-1/PD-L1 blockade (22, 23), and increased baseline thyroid FDG uptake may indicate subclinical thyroid inflammation, pre-existing autoimmune susceptibility, or early immune activation preceding overt biochemical or clinical thyroiditis. A potential concern is circularity, given that thyroiditis was the most frequent irAE in our cohort; however, sensitivity analysis excluding patients whose first documented irAE was thyroiditis preserved both discrimination and the independent contribution of thyroid SUVmax, indicating that the association extends beyond first-thyroiditis events alone.

NLR contributed information complementary to PET-derived immune-organ markers. An elevated NLR may reflect neutrophil-predominant inflammation, lymphocyte depletion, or imbalance between innate inflammatory activation and adaptive immune competence. Recent meta-analyses and cohort studies validate NLR as a robust predictor of irAEs and immunotherapy outcomes in NSCLC (24, 25). Our finding of an independent association indicates that systemic inflammation and organ-level FDG uptake reflect complementary dimensions of host immunity, advocating for their combined prognostic integration rather than mutual substitution.

An unexpected observation was that whole-body MTV was lower in patients who subsequently developed irAEs (*p* = 0.034). Several non-exclusive explanations are plausible. A lower tumour burden may correspond to a less immunosuppressive, tumour-derived metabolic and cytokine load (20), leaving the host immune system comparatively less exhausted and therefore more able to mount the vigorous—and potentially less self-tolerant—response that underlies irAEs (19); this would be consistent with the concurrently higher splenic and thyroidal FDG uptake and NLR observed in the same patients. It is equally important to note, however, that MTV was not an independent predictor on univariable analysis (OR 0.74 per 1-SD increase; *p* = 0.121) and was not retained in the final model, and that the borderline *p* value raises the possibility of a chance finding in a moderate-sized cohort. We therefore interpret this association cautiously and regard it as hypothesis-generating rather than as a robust mechanistic signal.

A central implication of these findings is that baseline PET/CT performed for oncologic staging contains underutilized toxicity-related information. Recent work has demonstrated that radiomic and quantitative PET features can predict both clinical benefit and toxicity from immunotherapy in NSCLC (26, 27). By relying on simple, reproducible, and interpretable variables rather than high-dimensional radiomics, the present model is well positioned for multicenter replication and real-world deployment.

Translating quantitative immune-organ metrics such as SLR and thyroid SUVmax into multicenter practice will require careful attention to measurement harmonization. Although interobserver agreement was high at our center, SUV-based metrics are sensitive to scanner hardware, reconstruction algorithms, uptake time, and plasma glucose, and liver normalisation (as used for SLR and BLR) only partially mitigates this variability. Cross-platform deployment will therefore depend on standardized acquisition and harmonization frameworks—such as EANM/EARL accreditation and the joint EANM/SNMMI/ANZSNM procedure standards for ^18^F-FDG PET during immunomodulatory treatment (9, 10)—together with prospective assessment of interobserver and interscanner reproducibility for these specific metrics. The need for standardized, cross-platform biomarker measurement is a recurring theme in lung-cancer immunotherapy biomarker development more broadly (28), and should be addressed explicitly before SLR and thyroid SUVmax can be adopted as generalisable risk-stratification tools.

Treatment heterogeneity is an additional consideration in interpreting these results. Patients received one of four anti-PD-1 agents, and 74.2% received anti-PD-1 combined with chemotherapy whereas 25.8% received monotherapy. Cytotoxic chemotherapy can independently alter splenic and bone-marrow metabolism and circulating leukocyte counts, and might therefore influence both the imaging predictors and the NLR. To address this, we included *post hoc* adjustment for treatment regimen and treatment line, after which the model retained strong discrimination (AUC 0.862) and all three predictors remained independently associated with irAEs (Supplementary Table S1). A formal subgroup analysis stratified by monotherapy versus combination therapy would be more informative, but the monotherapy subgroup (*n* = 31, 18 events) was too small to support stable multivariable estimation, whereas the combination subgroup (*n* = 89) dominates the cohort and its estimates closely track the overall model. Adequately powered analyses within treatment strata—and ideally within single-agent regimens—are therefore an important direction for future multicenter work.

We also considered established tumour-based biomarkers of immunotherapy response and toxicity. PD-L1 tumour proportion score was available for all patients and was included among the candidate clinical variables; however, it did not differ significantly between patients with and without irAEs (Table 1) and was not selected for the final model. Tumour mutational burden was not routinely assessed in clinical practice at our center during the study period and was therefore unavailable for most patients, precluding its inclusion. Because both markers are more closely linked to antitumour efficacy than to toxicity, and given the limited and inconsistent evidence relating them to irAE risk, their absence is unlikely to have materially biased the present toxicity-focused model; nevertheless, integrating PD-L1, tumour mutational burden, and other molecular features with immune-organ metabolic metrics is a worthwhile objective for future studies, particularly for the prediction of severe irAEs.

The absence of an OS difference after 3-month landmark correction is also clinically informative. Many studies have reported associations between irAEs and improved outcomes in NSCLC, but analyses based on ever-irAE status are vulnerable to immortal-time bias because patients must survive long enough to develop an irAE (29, 30). In our cohort, the landmark analysis revealed no detectable OS difference between patients with and without early irAEs. Accordingly, the primary value of the model lies in toxicity-risk stratification rather than prognostic prediction. Patients with higher baseline SLR, thyroid SUVmax, and NLR may benefit from intensified toxicity surveillance—including earlier symptom review, scheduled thyroid-function testing, and structured patient education on early irAE reporting. These markers should not be used to withhold anti-PD-1 therapy, in line with current guidance emphasizing early recognition rather than pre-emptive discontinuation in asymptomatic patients (4, 5).

Given the rising global burden of lung cancer and the rapid expansion of immune checkpoint inhibitor use across treatment lines (31), pretreatment risk-stratification tools that leverage routinely acquired imaging are clinically attractive. This study has several limitations. First, this is a single-center retrospective analysis with a moderate sample size of 120 patients and 66 irAE events, and external validation in independent multicenter cohorts is warranted before clinical application; although bootstrap internal validation reproduced the full model-development pipeline, performance estimates may remain optimistic. External validation in an independent, and preferably multicenter, cohort is therefore an indispensable prerequisite before any clinical application, and the optimism-corrected AUC of 0.850 should be regarded as an internally optimistic upper bound rather than as an estimate of out-of-sample performance. Second, the primary endpoint combined heterogeneous any-grade irAEs, and although a descriptive analysis of grade ≥3 events was provided, future studies with larger samples are needed to specifically address severe irAEs. Notably, when grade 3–4 events were used as the outcome, discrimination fell from an apparent AUC of 0.863 to 0.724 (Supplementary Table S1) and only thyroid SUVmax retained statistical significance, indicating that the model predicts the occurrence of any irAE more reliably than that of clinically severe irAEs—which remain the principal safety concern. The any-grade model should therefore be regarded as a trigger for intensified surveillance rather than as a validated predictor of severe toxicity. Third, despite standardized acquisition, residual variability in reconstruction protocols, together with potential confounding from subclinical thyroid pathology, treatment regimen, and concomitant medications, cannot be entirely excluded. Despite these limitations, our findings provide a clinically applicable, low-cost approach using imaging that is already routinely acquired before immunotherapy.

## Conclusion

Baseline SLR, thyroid SUVmax, and NLR were each independently associated with subsequent irAEs in patients with NSCLC receiving anti-PD-1 therapy, and their combination yielded a parsimonious, biologically coherent model with favorable apparent discrimination (AUC 0.863; optimism-corrected 0.850). These findings support a mechanistically grounded framework in which organ-level immune metabolism and systemic inflammation jointly reflect host susceptibility to immune toxicity, and they highlight the previously underutilized role of staging PET/CT as a source of toxicity-relevant information. Pending prospective multicenter validation, recalibration, and net-benefit assessment, this approach has the potential to support PET/CT-informed irAE risk stratification and enable more individualized safety monitoring during anti-PD-1 therapy.

## Data Availability

The original contributions presented in the study are included in the article/Supplementary material, further inquiries can be directed to the corresponding author.

## References

[1] Reck M Remon J Hellmann MD. First-line immunotherapy for non-small-cell lung Cancer. J Clin Oncol. (2022) 40:586–97. doi: 10.1200/JCO.21.01497, 34985920

[2] Mok TSK Wu YL Kudaba I Kowalski DM Cho BC Turna HZ . Pembrolizumab versus chemotherapy for previously untreated, PD-L1-expressing, locally advanced or metastatic non-small-cell lung cancer (KEYNOTE-042): a randomised, open-label, controlled, phase 3 trial. Lancet. (2019) 393:1819–30. doi: 10.1016/S0140-6736(18)32409-7, 30955977

[3] Postow MA Sidlow R Hellmann MD. Immune-related adverse events associated with immune checkpoint blockade. N Engl J Med. (2018) 378:158–68. doi: 10.1056/NEJMra1703481, 29320654

[4] Schneider BJ Naidoo J Santomasso BD Lacchetti C Adkins S Anadkat M . Management of Immune-Related Adverse Events in patients treated with immune checkpoint inhibitor therapy: ASCO guideline update. J Clin Oncol. (2021) 39:4073–126. doi: 10.1200/JCO.21.01440, 34724392

[5] Haanen J Obeid M Spain L Carbonnel F Wang Y Robert C . Management of toxicities from immunotherapy: ESMO clinical practice guideline for diagnosis, treatment and follow-up. Ann Oncol. (2022) 33:1217–38. doi: 10.1016/j.annonc.2022.10.001 36270461

[6] Su J Li Y Tan S Cheng T Luo Y Zhang L. Pretreatment neutrophil-to-lymphocyte ratio is associated with immunotherapy efficacy in patients with advanced cancer: a systematic review and meta-analysis. Sci Rep. (2025) 15:446. doi: 10.1038/s41598-024-84890-3, 39747391 PMC11695637

[7] Zhang W Tan Y Li Y Liu J. Neutrophil to lymphocyte ratio as a predictor for immune-related adverse events in cancer patients treated with immune checkpoint inhibitors: a systematic review and meta-analysis. Front Immunol. (2023) 14:1234142. doi: 10.3389/fimmu.2023.1234142, 37622124 PMC10445236

[8] Aide N Hicks RJ Le Tourneau C Lheureux S Fanti S Lopci E. FDG PET/CT for assessing tumour response to immunotherapy: report on the EANM symposium on immune modulation and recent review of the literature. Eur J Nucl Med Mol Imaging. (2019) 46:238–50. doi: 10.1007/s00259-018-4171-4, 30291373 PMC6267687

[9] Boellaard R Delgado-Bolton R Oyen WJ Giammarile F Tatsch K Eschner W . European Association of Nuclear Medicine (EANM). FDG PET/CT: EANM procedure guidelines for tumour imaging: version 2.0. Eur J Nucl Med Mol Imaging. (2015) 42:328–54. doi: 10.1007/s00259-014-2961-x, 25452219 PMC4315529

[10] Lopci E Hicks RJ Dimitrakopoulou-Strauss A Dercle L Iravani A Seban RD . Joint EANM/SNMMI/ANZSNM practice guidelines/procedure standards on recommended use of [^18^F]FDG PET/CT imaging during immunomodulatory treatments in patients with solid tumors version 1.0. Eur J Nucl Med Mol Imaging. (2022) 49:2323–41. doi: 10.1007/s00259-022-05780-2, 35376991 PMC9165250

[11] Karlsen W Akily L Mierzejewska M Teodorczyk J Bandura A Zaucha R . Is ^18^F-FDG-PET/CT an optimal imaging modality for detecting immune-related adverse events after immune-checkpoint inhibitor therapy? Pros and cons. Cancers (Basel). (2024) 16:1990. doi: 10.3390/cancers16111990, 38893111 PMC11171385

[12] Santo G Cucè M Restuccia A Del Giudice T Tassone P Cicone F . Immune-related [18F]FDG PET findings in patients undergoing checkpoint inhibitors treatment: correlation with clinical adverse events and prognostic implications. Cancer Imaging. (2024) 24:125. doi: 10.1186/s40644-024-00774-9, 39289716 PMC11409779

[13] Collins GS Reitsma JB Altman DG Moons KG. Transparent reporting of a multivariable prediction model for individual prognosis or diagnosis (TRIPOD): the TRIPOD statement. BMJ. (2015) 350:g7594–4. doi: 10.1136/bmj.g7594, 25569120

[14] Collins GS Moons KGM Dhiman P Riley RD Beam AL Van Calster B . TRIPOD+AI statement: updated guidance for reporting clinical prediction models that use regression or machine learning methods. BMJ. (2024) 385:e078378. doi: 10.1136/bmj-2023-078378, 38626948 PMC11019967

[15] Wahl RL Jacene H Kasamon Y Lodge MA. From RECIST to PERCIST: evolving considerations for PET response criteria in solid tumors. J Nucl Med. (2009) 50 Suppl 1:122S–50S. doi: 10.2967/jnumed.108.057307, 19403881 PMC2755245

[16] Riley RD Snell KI Ensor J Burke DL Harrell FE Jr Moons KG . Minimum sample size for developing a multivariable prediction model: PART II—binary and time-to-event outcomes. Stat Med. (2019) 38:1276–96. doi: 10.1002/sim.7992, 30357870 PMC6519266

[17] Jin P Bai M Liu J Yu J Meng X. Tumor metabolic and secondary lymphoid organ metabolic markers on 18F-fludeoxyglucose positron emission tomography predict prognosis of immune checkpoint inhibitors in advanced lung cancer. Front Immunol. (2022) 13:1004351. doi: 10.3389/fimmu.2022.1004351, 36341372 PMC9634068

[18] Madsen AM Gerke O Ruhlmann CH Hildebrandt MG Nadaraja S. The prognostic value of biomarkers identified by [^18^F]FDG-PET/CT in patients with high-risk melanoma treated with adjuvant immunotherapy. Diagnostics (Basel). (2025) 16:79. doi: 10.3390/diagnostics16010079, 41515575 PMC12785806

[19] Wei SC Duffy CR Allison JP. Fundamental mechanisms of immune checkpoint blockade therapy. Cancer Discov. (2018) 8:1069–86. doi: 10.1158/2159-8290.CD-18-0367, 30115704

[20] Veglia F Sanseviero E Gabrilovich DI. Myeloid-derived suppressor cells in the era of increasing myeloid cell diversity. Nat Rev Immunol. (2021) 21:485–98. doi: 10.1038/s41577-020-00490-y, 33526920 PMC7849958

[21] Galligan A Wallace R Krishnamurthy B Kay TWH Sachithanandan N Chiang C . Increased thyroidal activity on routine FDG-PET/CT after combination immune checkpoint inhibition: temporal associations with clinical and biochemical thyroiditis. Cancers (Basel). (2023) 15:5803. doi: 10.3390/cancers15245803, 38136348 PMC10741830

[22] Muir CA Clifton-Bligh RJ Long GV Scolyer RA Lo SN Carlino MS . Thyroid immune-related adverse events following immune checkpoint inhibitor treatment. J Clin Endocrinol Metab. (2021) 106:e3704–13. doi: 10.1210/clinem/dgab263, 33878162

[23] Iyer PC Cabanillas ME Waguespack SG Hu MI Thosani S Lavis VR . Immune-related thyroiditis with immune checkpoint inhibitors. Thyroid. (2018) 28:1243–51. doi: 10.1089/thy.2018.0116, 30132401 PMC6157359

[24] Lu HR Zhu PF Deng YY Chen ZL Yang L. Predictive value of NLR and PLR for immune-related adverse events: a systematic review and meta-analysis. Clin Transl Oncol. (2024) 26:1106–16. doi: 10.1007/s12094-023-03313-3, 37682501

[25] Pavan A Calvetti L Dal Maso A Attili I Del Bianco P Pasello G . Peripheral blood markers identify risk of immune-related toxicity in advanced non-small cell lung Cancer treated with immune-checkpoint inhibitors. Oncologist. (2019) 24:1128–36. doi: 10.1634/theoncologist.2018-0563, 31015312 PMC6693718

[26] Mu W Tunali I Gray JE Qi J Schabath MB Gillies RJ. Radiomics of ^18^F-FDG PET/CT images predicts clinical benefit of advanced NSCLC patients to checkpoint blockade immunotherapy. Eur J Nucl Med Mol Imaging. (2020) 47:1168–82. doi: 10.1007/s00259-019-04625-9, 31807885 PMC8663718

[27] Kim KH Kim SM Lee JE Kim SS Kang DH Chung C. Predictive role of baseline peripheral lung SUVmax on PET/CT for immune-related pneumonitis and adverse events in lung Cancer patients treated with immune checkpoint inhibitors. Cancer Res Treat. (2025). doi: 10.4143/crt.2025.548, 41166927

[28] Ji K Guo L Zuo D Feng M Chen X Zhao Z . Harnessing delta-like ligand 3: bridging biomarker discovery to next-generation immunotherapies in refractory small cell lung cancer. Front Immunol. (2025) 16:1592291. doi: 10.3389/fimmu.2025.1592291, 40496850 PMC12149116

[29] Hussaini S Chehade R Boldt RG Raphael J Blanchette P Maleki Vareki S . Association between immune-related side effects and efficacy and benefit of immune checkpoint inhibitors—a systematic review and meta-analysis. Cancer Treat Rev. (2021) 92:102134. doi: 10.1016/j.ctrv.2020.102134, 33302134

[30] Petrelli F Grizzi G Ghidini M Ghidini A Ratti M Panni S . Immune-related adverse events and survival in solid tumors treated with immune checkpoint inhibitors: a systematic review and Meta-analysis. J Immunother. (2020) 43:1–7. doi: 10.1097/CJI.0000000000000300, 31574022

[31] Sung H Ferlay J Siegel RL Laversanne M Soerjomataram I Jemal A . Global Cancer statistics 2020: GLOBOCAN estimates of incidence and mortality worldwide for 36 cancers in 185 countries. CA Cancer J Clin. (2021) 71:209–49. doi: 10.3322/caac.21660, 33538338

