# Supplementary material for: Baseline immune-organ 18F-FDG PET/CT metabolism is associated with immune-related adverse events in anti-PD-1-treated non-small cell lung cancer
Source: Front Med (Lausanne). 2026 Aug 26;13:1920274. doi: 10.3389/fmed.2026.1920274 (PMC13553489; doi:10.3389/fmed.2026.1920274)
Supplement: Supplementary file 1 [file table_1.docx]

**Supplementary Table S1. Sensitivity analyses**

| **Analysis** | **N** | **Events** | **AUC** | **SLR OR (95% CI)** | **P** | **Thyroid OR (95% CI)** | **P** | **NLR OR (95% CI)** | **P** |
| --- | --- | --- | --- | --- | --- | --- | --- | --- | --- |
| Excluding thyroiditis as first irAE | 100 | 46 | 0.874 | 3.97 (2.14–7.36) | <0.001 | 3.63 (1.92–6.87) | <0.001 | 2.12 (1.26–3.58) | 0.005 |
| Grade 3–4 irAE vs all others | 120 | 18 | 0.724 | 1.58 (0.95–2.63) | 0.075 | 1.89 (1.10–3.25) | 0.022 | 1.56 (0.92–2.64) | 0.097 |
| Adjusted for age, sex, ECOG PS | 120 | 66 | 0.875 | 4.28 (2.29–8.00) | <0.001 | 3.84 (2.08–7.08) | <0.001 | 2.08 (1.25–3.48) | 0.005 |
| Adjusted for Tx regimen/line | 120 | 66 | 0.862 | 4.44 (2.38–8.29) | <0.001 | 3.71 (1.99–6.92) | <0.001 | 2.00 (1.23–3.24) | 0.005 |

**Note:** All models used the same three standardized predictors (SLR, thyroid SUVmax, NLR) from the primary analysis. The first row excludes patients whose first documented irAE was thyroiditis to assess whether the thyroid SUVmax association was driven by pre-existing subclinical thyroid disease. The second row redefines the outcome as grade ≥3 irAEs. The third and fourth rows include additional covariates.

**Abbreviations:** AUC, area under the receiver operating characteristic curve; CI, confidence interval; ECOG PS, Eastern Cooperative Oncology Group performance status; irAE, immune-related adverse event; NLR, neutrophil-to-lymphocyte ratio; OR, odds ratio; SLR, spleen-to-liver ratio; SUV, standardized uptake value; Tx, treatment.
